# Telocytes and Lymphatics of the Human Colon

**DOI:** 10.3390/life11101001

**Published:** 2021-09-23

**Authors:** Mihai Zurzu, Mihnea Ioan Nicolescu, Laurențiu Mogoantă, Stelian Pantea, Mugurel Constantin Rusu

**Affiliations:** 1 Division of Anatomy, Faculty of Dental Medicine, “Carol Davila” University of Medicine and Pharmacy, 050474 Bucharest, Romania; mihai.zurzu@drd.umfcd.ro; 2 Division of Histology, Faculty of Dental Medicine, “Carol Davila” University of Medicine and Pharmacy, 050474 Bucharest, Romania; 3 Laboratory of Radiobiology, “Victor Babeș” National Institute of Pathology, 050096 Bucharest, Romania; 4 Department of Histology, University of Medicine and Pharmacy Craiova, 200349 Craiova, Romania; laurentiu.mogoanta@umfcv.ro; 5 Surgery Clinic II, “Victor Babeș” University of Medicine and Pharmacy, 300041 Timișoara, Romania; pantea.stelian@umft.ro

**Keywords:** interstitial Cajal cells, CD34, podoplanin, gastrointestinal tract

## Abstract

Background: Telocytes (TCs) are a peculiar morphological type of stromal cells. They project long and moniliform telopodes, visible on various bidimensional sections. Originally regarded as “interstitial Cajal-like cells”, gastrointestinal TCs were CD34+. Further double-labelling studies found that colon TCs are negative for the expressions of the PDGFR-α and α-SMA. However, the TCs in colon were not distinguished specifically from endothelial cells (ECs), vascular or lymphatic. A combinational approach is important for accurate TC identification. Hence, we designed an immunohistochemical study of human colon to check whether ECs and CD34+ TCs express different markers. Methods: Immunohistochemistry was performed on archived paraffin-embedded samples of human colon (nine cases) for the following markers: CD31, CD34, CD117/c-kit and D2-40 (podoplanin). Results: A distinctive population of CD34+ TCs was found coating the myenteric ganglia. However, also perivascular cells and vascular ECs were CD34+. c-kit expression was equally found in interstitial Cajal cells (ICCs) and perivascular cells. The CD34 TCs did not express c-kit. As they were equally CD31- and D2-40- they were assessed as different from ECs. Conclusions: Testing specific markers of ECs, vascular and lymphatic, in the same tissues in which CD34+ TCs are found, is much more relevant than to identify TCs by transmission electron microscopy alone.

## 1. Introduction

In 2008, Pieri et al. used a CD117/c-kit and CD34 to study the interstitial cells of the human gut wall [1]. Their study used light and transmission electron microscopy and they claimed to find a type of cells different from interstitial Cajal cells (ICCs) or any other possible cell type known at that time. Therefore, they termed the cells they found “interstitial Cajal-like cells” (ICLCs). They identified septal ICLCs, ICLCs in the myenteric plexus, as well as ICLCs bordering the muscle layers [1]. However, only the expression of CD34 was verified by immunoelectron microscopy (IEM). Such immunoelectron microscopy studies are still lacking in the study of TCs and should be used to identify both the ultrastructural traits and the immunophenotype of TCs [2].

Two years later, Popescu and Faussone-Pellegrini proposed in an Editorial Material [3] a new cell type and renamed the ICLCs as “telocytes” (TCs). The ultrastructural evidence of an interstitial CD34+ cell with a thin prolongation from the wall of the small intestine previously reported (Figure 6E in Pieri et al. [1]) was then reconsidered as being a TC (Figure 13 in Popescu and Faussone-Pellegrini [3]). TCs were there defined by a peculiar morphological pattern: cells with thin prolongations, which were soon termed telopodes. The telopodes consisted in thin segments (podomeres) and dilations (podoms) and further served in numerous studies [4,5,6,7] to identify TCs. However, identification of TCs on just a morphological basis seems unreliable [8,9,10]. On the other hand, the IEM initial evidence of CD34+ ICLCs/TCs [1] still stands alone and further immunohistochemical studies led to false tracks [11,12,13], also due to the fact that initial studies of TCs failed to differentiate TCs from lymphatic endothelial cells [14]. Recently, a study on human eye conjunctiva took into account the negative expression of endothelial (CD31) and lymphatic (podoplanin) markers in TCs [15]. Therefore, a CD31-/CD34+/podoplanin phenotype could be indicative for identifying TCs in light microscopy. Since CD34 is also expressed in cells of the hematopoietic lineage, this could represent another weakness of precise TC diagnosis [16].

A combinational approach is important for the improvement of TC identification. Each method provides unique benefits and unfortunately also disadvantages. Brightfield microscopy is able to yield valuable histological information that are replaced by other kind of optical data in fluorescence microscopy for example, where the advantages of double-labelling prevail. That is why important to choose the most suitable methodology related to the scientific scope of the problem at hand. Thus, we decided on an immunohistochemical study of human colon to check whether the CD34+ ICLCs/TCs are immunophenotypically distinct of endothelial cells (ECs), either vascular or lymphatic.

## 2. Materials and Methods

The immunohistochemical study was performed retrospectively on archived paraffin-embedded samples of human colon (nine cases). The ages of donor patients ranged from 58 to 66 years. The patients’ signed informed consent for all medical data to be used for research purposes, provided that the protection of their identity is maintained, foreran tissues processing. The study was tacitly approved by the responsible authorities where the work was carried out, and it was conducted in accordance with the general principles of medical research, as stated in the Declaration of Helsinki. All the procedures were followed by the Institutional Ethics Committee of the University of Medicine and Pharmacy Craiova (ref. no. 74/11.07.2016).

The paraffin-embedded samples were processed as previously [13] with an automatic histoprocessor (Diapath, Martinengo, BG, Italy). Sections were cut at 3 μm and mounted on SuperFrost^®^ electrostatic slides for immunohistochemistry (Thermo Scientific, Menzel-Gläser, Braunschweig, Germany). Histological evaluations used 3 μm-thick sections stained with hematoxylin and eosin. Internal negative controls resulted when the primary antibodies were not applied on slides.

We used primary antibodies for CD34 (Cat# CM 084 A, B, C, clone QBEnd/10, Biocare Medical, Concord, CA, USA, 1:50), D2-40 (Cat# CM 266 A, B, C, clone D2-40, Biocare Medical, 1:100), CD117/c-kit (Cat# CME 296 CK, RRID:AB_10581361, clone Y145, Biocare Medical, 1:100) and CD31 (PECAM-1) (Cat# CM 347 A, C, clone BC2, Biocare Medical, 1:200).

Tissues were deparaffinized and rehydrated; then endogenous peroxidase was blocked using Peroxidazed 1 (Biocare Medical). For the heat-induced epitope retrieval, we used the Decloaking Chamber (Biocare Medical) and retrieval solution pH 6 (Biocare Medical). We used Background Blocker (Biocare Medical) to reduce nonspecific background staining. The primary antibody was then applied. We used different HRP-based detection systems: for CD31 and CD117 we used MACH 2 (Biocare Medical), for D2-40 we used MACH 4 (Biocare Medical), and for CD34 the two-steps detection used a 4 plus detection system. A HRP-compatible chromogen (DAB) was applied. Sections were counterstained with hematoxylin and rinsed with deionized water. For the washing steps, we used TBS solution, pH 7.6.

## 3. Results

**Figure 1:**
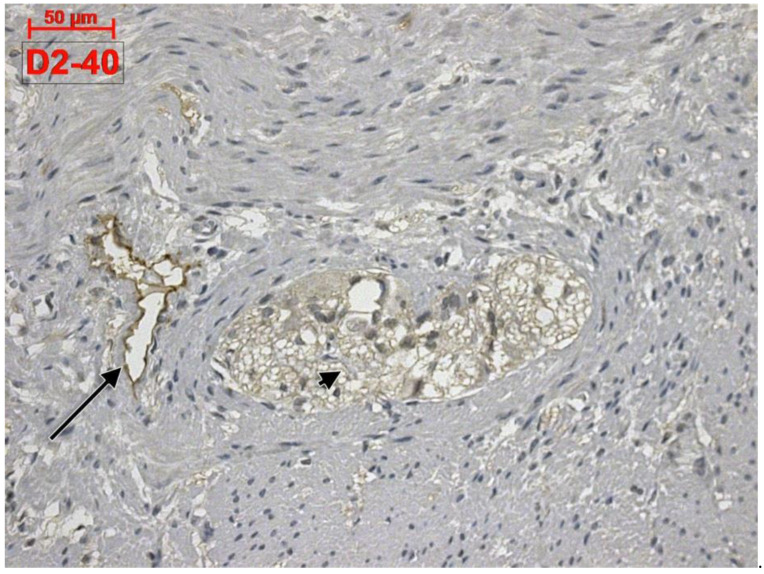
Human colon. D2-40 is exclusively expressed in the lymphatics of colon. A myenteric ganglion (arrowhead) and a neighbor lymphatic lacuna (arrow) are located within the muscularis externa.

**Figure 2:**
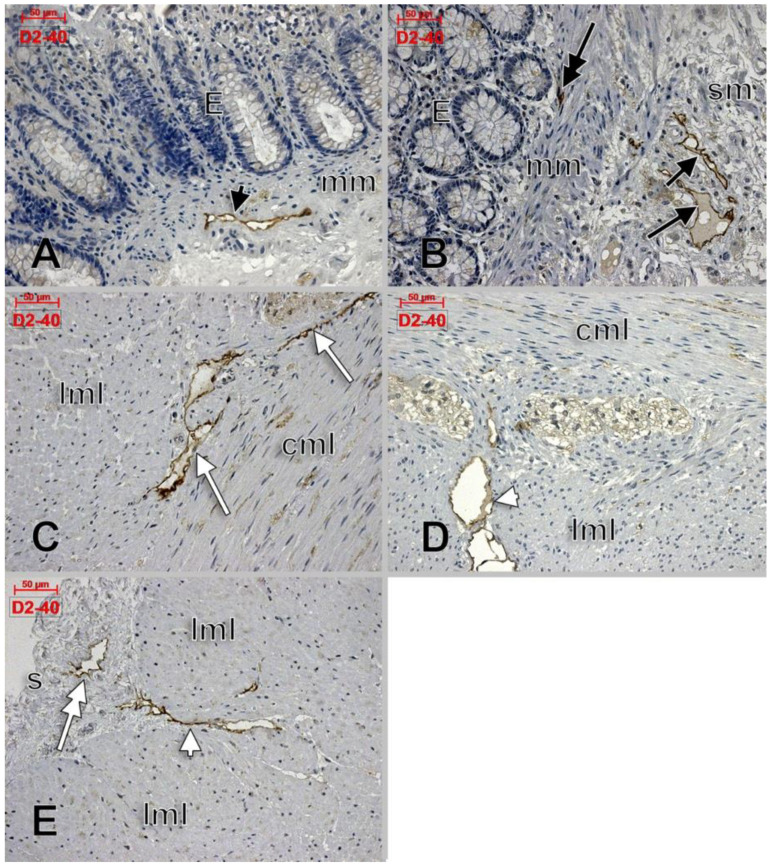
D2-40-labeled lymphatics of the colon wall in several regions: muscularis mucosae (**A**, black arrowhead), subepithelial (**B**, double-headed black arrow), submucosa (**B**, black arrows), circumferential lymphatics of the muscularis propria, between the circular and longitudinal muscle layers (**C**, white arrows), transverse lymphatics of the longitudinal muscle layer (**D**,**E**, white arrowheads), and subserosal lymphatics (**E**, double-headed arrow). E: epithelium; mm: muscularis mucosae; sm: submucosa; cml: circular muscle layer; lml: longitudinal muscle layer; s: serosa.

**Figure 3:**
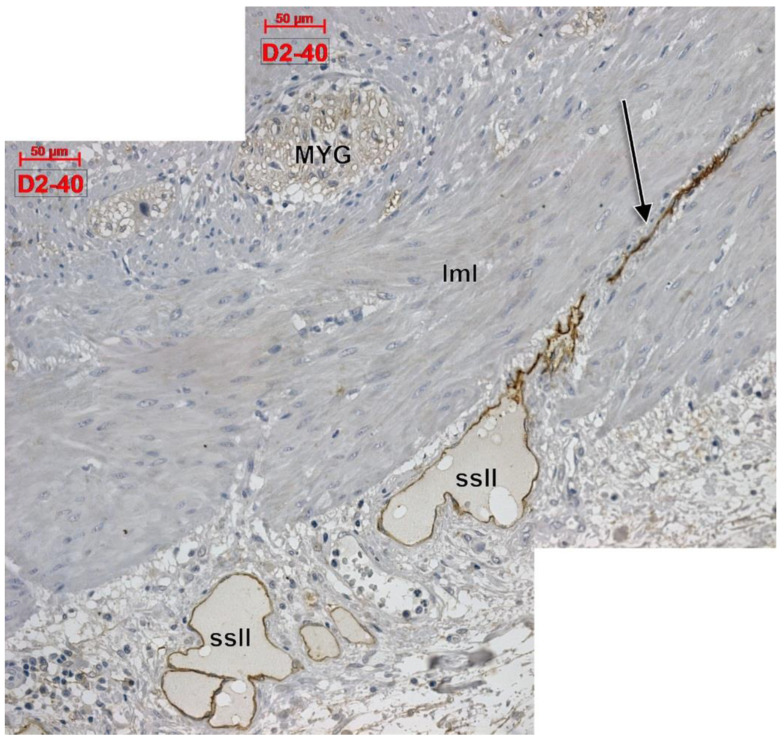
Longitudinal cut of the outer colon wall, immune expression of D2-40. MYG: myenteric ganglion; lml: longitudinal muscle layer; ssll: subserosal lymphatic lacunae; arrow: longitudinal lymphatic.

Apparently, D2-40 exclusively labelled lymphatics of the colon wall (Figure 1 and Figure 2). We found subepithelial lymphatics exclusively located on the muscularis mucosae, lymphatics of the muscularis mucosae, submucosal lymphatics located beneath the muscularis mucosae or applied on the circular muscle layer, as well as lymphatics of the muscularis externa: (a) transverse lymphatics of the circular muscle layer; (b) circumferential lymphatics and lymphatic lacunae placed in the myenteric layer between the circular and longitudinal muscle layers; (c) lymphatic lacunae and transversal and longitudinal lymphatics in the outer longitudinal layer. Finally, we encountered subserosal lymphatics and lymphatic lacunae (Figure 2 and Figure 3).

**Figure 4:**
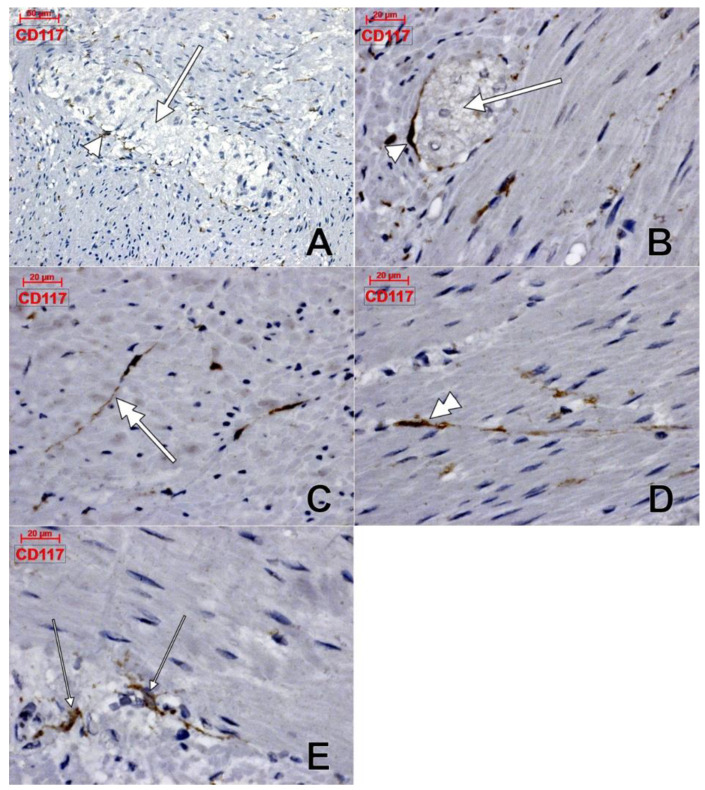
Human colon, immune histochemistry of CD117/c-kit. Myenteric ganglia (**A**,**B**, thick arrows) are surrounded by scarce c-kit+ cells (**A**,**B**, arrowheads). c-kit+ telocyte-like cells are equally located within the longitudinal muscle (**C**, double-headed arrow), and circular (**D**, double-headed arrowhead) layers. Other c-kit+ cells (**E**, thin arrows) are also present in the myenteric layer.

**Figure 5:**
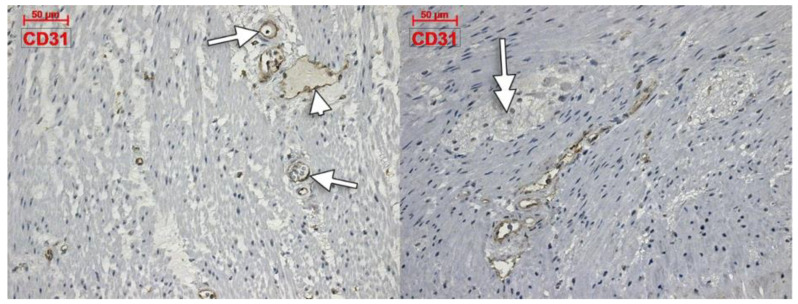
Immune expression of CD31 in the colon muscularis propria is restricted to vascular (arrows) and lymphatic (arrowhead) endothelial cells. No labelling is observed within and around the myenteric ganglia (double-headed arrow).

**Figure 6:**
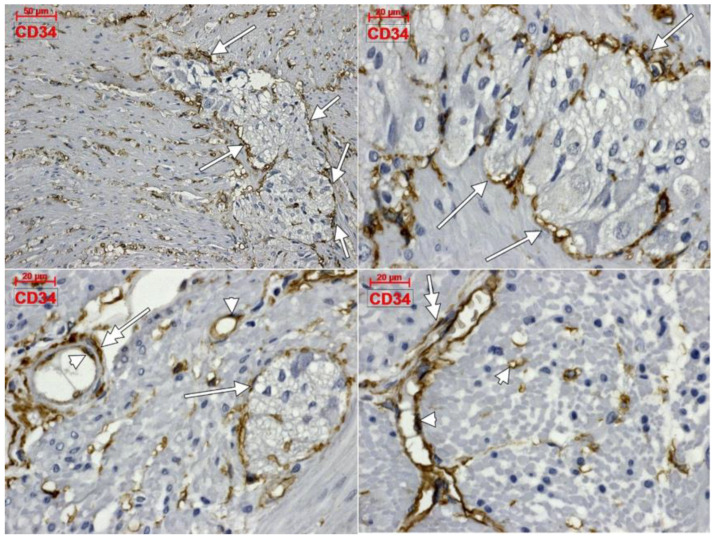
Immune expression of CD34 in the human colon. The antigen is expressed in complete coats of the myenteric ganglia (arrows), endothelial cells (arrowheads), as well as in perivascular cells (double-headed arrows).

Within the muscularis propria we found scarce c-kit-expressing cells bordering myenteric ganglia (Figure 4). No c-kit immunoreactivity was observed within the myenteric ganglia. We also identified c-kit+ spindle-shaped TC-like cells within the circular, and longitudinal, muscle layers (Figure 4). In the stroma of the myenteric layer, microvessels were found covered by c-kit+ cell, that could be mast cells or pericytes, but their morphology leans toward the latter (Figure 4). ECs did not express c-kit. Within the muscularis propria, expression of CD31 was exclusively found in ECs–both vascular and lymphatic (Figure 5). CD34+ cells completely surrounded the myenteric ganglia. The marker was also expressed in vascular ECs and in perivascular cells within the septa of the muscularis propria (Figure 6).

## 4. Discussion

The gastrointestinal tract is an organ system which critically depends on adequate lymphatic functioning [17]. However, only a few studies have examined the gastrointestinal lymphatic structure, regulation and functioning [17]. It was established that lymphatic remodeling occurs during active inflammatory bowel diseases, such as Crohn’s disease and ulcerative colitis [18]. This could be related with the expression of VEGF-C, the lymphangiogenic factor, in normal colon tissues [19].

The intramural lymphatics of the colon were studied in rabbits, rats and guinea pigs [20]. The authors found lymphatic vessels in the submucosa, muscularis propria and subserosa [20]. They also reported lymphatic capillaries in the mucosa [20]. The function of intramucosal colon lymphatics is unknown, but they may facilitate metastatic spread in the event of a malignant tumor [19]. This repartition corresponds to that one in the canine colon [21]. In the canine colon, the lymphatics of lamina propria form a shallow layer beneath the epithelium and a deep one above the muscularis mucosae [22]. The vessels draining from the muscularis propria into the subserosa run parallel to the intestinal axis [22]. To the authors’ knowledge, evidence demonstrating the anatomy of the human colon intramural lymphatics was not previously brought.

Dilated lymphatic spaces lined with a simple layer of endothelial cells were described in uterine tubes as “lymphatic lacunae” in English and as “lacunae lymphaticae” in Latin [23]. We found here lymphatic lacunae of the colon wall: submucosal, myenteric and subserosal. It is therefore reasonable to speculate a pump mechanism in which the muscular tissue of the colon wall pumps the lymph into these layered lymphatic lacunae.

Thomsen et al. presented ultrastructural evidence of a part of an isolated mouse ICC from a four-day culture, focused on one cell process [24]. The authors considered at that time they found “a unique combination of ultrastructural characteristics” consisting of plasmalemmal caveolae, electron-dense mitochondria and an aligned group of intermediate filaments within that cell process [24]. Interestingly, such characteristics were equally found in human adipose-derived mesenchymal stem cells (MSCs) [25]. Therefore, they could not be regarded as specific ultrastructural traits of ICCs.

“Isolated external muscle layers” of intestine were processed to further isolate smooth muscle cells and ICCs from the myenteric plexus area in different experiments [24,26]. The authors overlooked the vascular and stromal perivascular cells within those muscle layers, which we demonstrated here in light microscopy using c-kit and CD34 labeling. Therefore, further cultured cells could’ve equally been perivascular, or vascular cells.

Experiments [26] left from previous evidence of ICCs being “the only cells in the gut that are double positive for c-kit and CD34”. We carefully reviewed that evidence which was focused mainly on the gastrointestinal stromal tumors [27,28], as also Pieri et al. previously observed [1].

In one of those studies, CD34 expression was detected in interstitial cells surrounding myenteric ganglia and this was only “suggestive” for a subpopulation of CD34+ ICCs [27]. In that study [27] CD34+ and, respectively, c-kit+ cells were coating the myenteric ganglia, such as we also found here. Same as we found in the present study, c-kit+ ICCs were located between the circular and longitudinal layers of the muscularis propria and were either associated, or were enclosing myenteric ganglia [27]. However, this is not an exclusive location, as we demonstrated here also c-kit+ ICCs present within the longitudinal and circular muscle layers. Moreover, we found that c-kit+ ICCs do not form a complete coat of the myenteric ganglia, while CD34+ cells build complete ganglionic coats. Therefore, if a stromal cell is CD34+, it should not be regarded by default as a c-kit+ ICC. In another study [28] co-expression of CD34 and c-kit was equally found in gastrointestinal stromal tumors and around the myenteric ganglia in normal tissues. Fibroblast-like cells were found expressing CD34 but they were c-kit-negative [28]. These could correspond to the CD34+ perivascular cells we found here within the myenteric layer between the circular and longitudinal muscle layers. Indeed, CD34 is not co-expressed in c-kit+ ICCs in the gut [29].

Identification of CD34+ cells resembling the ICCs was attempted by Pieri et al. in 2008 [1]. Although they indicated the use of “ascending and descending colon” samples, they only presented evidence of: (a) CD34 expression in the submucosa of the large intestine; (b) CD34+ nerve sheaths in the large intestine; (c) TEM evidence of ICLCs covering myenteric ganglia in the large intestine [1]. For TEM, “full-thickness strips of the muscle coat” and, further, “full-thickness thin sections of the muscle coat” were processed [1]. Although proofs of the c-kit expression were brought, they were not specific for the large intestine. Double labelling for c-kit and CD34 was demonstrated just for the gastric wall; it was presented evidence for closely apposed CD34+ and, respectively, c-kit+ cells [1]. The authors observed that “CD34-positive cells run in rows intercalated with the c-kit-positive cells” [1] which is, in our opinion, quite suggestive for longitudinally cut CD34 endothelial tubes, on which pericytes could have been applied. We found in the present study c-kit+ pericytes applied on CD34+ vascular ECs, none of these being either ICCs, or ICLCs/telocytes. Two years after that study, the ICLCs were renamed as TCs, which were assigned a CD34+ phenotype [3].

When TCs were characterized in the human gastrointestinal tract they were considered showing a CD34+/PDGFRα- immunohistochemical phenotype [30]. Thus they were distinguished from other interstitial cell types: ICCs and fibroblast-like cells [30]. However, they were not specifically distinguished from cells in the vascular niches, ECs and pericytes.

A previous histological and immunohistochemical study aimed at observing the peculiarities of TCs in the normal and pathological colonic wall using CD34 and α-smooth muscle actin reported that CD34+ TCs were different from myoid cell types, such as myofibroblasts, pericytes and vascular smooth muscle cells [31]. In the afore-mentioned study the distinction between TCs and pericytes (both CD34+) was particularly made by the myoid phenotype and not by the c-kit expression.

We are aware of two medium limitations of this study–one being the small sample size and the other being the single-labelling technique in our experiments. Briefly, this study is focused on yielding information that should be taken together with results from other (complimentary) research methods. Hence, we chose from the beginning to use the full histological advantages given by brightfield microscopy and focus on peculiar cellular aspects depicted in the figures. This is how we were able to find in our current study colon c-kit+ cells that might be either pericytes or mast cells, either way making this marker unreliable to distinguish TCs from ICCs alone. A further double-fluorescence labelling would probably discriminate further, but we considered that this should be part of a different approach on the matter and was not included in this study.

## 5. Conclusions

At the time when TCs in the gastrointestinal tract were studied, neither CD31 nor podoplanin were used to discriminate ECs, vascular or lymphatic. Our results confirm that CD34+TCs in the colon wall are different of lymphatic ECs, labeled with CD31, as well as with D2-40, and of vascular ECs expressing CD31. Therefore, TEM alone is not so reliable to distinguish TCs from other cell types and it should be either used complementary with specific antibodies or discarded if it is intended as a unique method to identify TCs.

## Data Availability

The data presented in this study are available on request from the corresponding author upon reasonable request.
